# Impact of the Introduction of Rotavirus Vaccine on Hospital Admissions for Diarrhea Among Children in Kenya: A Controlled Interrupted Time-Series Analysis

**DOI:** 10.1093/cid/ciz912

**Published:** 2019-09-23

**Authors:** Grieven P Otieno, Christian Bottomley, Sammy Khagayi, Ifedayo Adetifa, Mwanajuma Ngama, Richard Omore, Billy Ogwel, Betty E Owor, Godfrey Bigogo, John B Ochieng, Clayton Onyango, Jane Juma, Jason Mwenda, Collins Tabu, Jacqueline E Tate, Yaw Addo, Tuck Britton, Umesh D Parashar, Robert F Breiman, Jennifer R Verani, D James Nokes

**Affiliations:** 1 Kenya Medical Research Institute (KEMRI)–Wellcome Trust Research Programme, Centre for Geographic Medicine Research-Coast, Kilifi, Kenya; 2 London School of Hygiene and Tropical Medicine, London, United Kingdom; 3 KEMRI–Centre for Global Health Research, Kisumu, Kenya; 4 Department of Pediatrics and Child Health, College of Medicine University of Lagos, Lagos, Nigeria; 5 Centers for Disease Control and Prevention–Kenya, Nairobi, Kenya; 6 World Health Organization Regional Office for Africa, Brazzaville, Republic of Congo; 7 Ministry of Health, Nairobi, Kenya; 8 Centers for Disease Control and Prevention, Emory University, Atlanta, Georgia, USA; 9 Emory Global Health Institute, Emory University, Atlanta, Georgia, USA; 10 School of Life Sciences and Zeeman Institute for Systems Biology and Infectious Disease Epidemiology Research, University of Warwick, Coventry, United Kingdom

**Keywords:** rotavirus vaccine, interrupted time series, control, vaccine impact

## Abstract

**Background:**

Monovalent rotavirus vaccine, Rotarix (GlaxoSmithKline), was introduced in Kenya in July 2014 and is recommended to infants as oral doses at ages 6 and 10 weeks. A multisite study was established in 2 population-based surveillance sites to evaluate vaccine impact on the incidence of rotavirus-associated hospitalizations (RVHs).

**Methods:**

Hospital-based surveillance was conducted from January 2010 to June 2017 for acute diarrhea hospitalizations among children aged <5 years in 2 health facilities in Kenya. A controlled interrupted time-series analysis was undertaken to compare RVH pre– and post–vaccine introduction using rotavirus-negative cases as a control series. The change in incidence post–vaccine introduction was estimated from a negative binomial model that adjusted for secular trend, seasonality, and multiple health worker industrial actions (strikes).

**Results:**

Between January 2010 and June 2017 there were 1513 and 1652 diarrhea hospitalizations in Kilifi and Siaya; among those tested for rotavirus, 28% (315/1142) and 23% (197/877) were positive, respectively. There was a 57% (95% confidence interval [CI], 8–80%) reduction in RVHs observed in the first year post–vaccine introduction in Kilifi and a 59% (95% CI, 20–79%) reduction in Siaya. In the second year, RVHs decreased further at both sites, 80% (95% CI, 46–93%) reduction in Kilifi and 82% reduction in Siaya (95% CI. 61–92%); this reduction was sustained at both sites into the third year.

**Conclusions:**

A substantial reduction in RVHs and all-cause diarrhea was observed in 2 demographic surveillance sites in Kenya within 3 years of vaccine introduction.

## Teaser

Following the national introduction of the rotavirus vaccine in Kenya, our impact evaluation across 2 surveillance sites indicates a substantial reduction in childhood hospitalization due to rotavirus-associated and all-cause severe diarrhea.

(See the Major Article by Khagayi et al on pages 2298–305 and Editorial Commentary by Steele and Groome, on pages 2314–6.)

Rotavirus is a major contributor to severe diarrhea illness and related mortality, especially in low- and middle-income countries [1–3]. Global estimates suggest that, in 2013, rotavirus accounted for 215 000 or 3.4% of all deaths in children aged less than 5 years [4]. In 2009, the World Health Organization recommended that all countries, especially those with high diarrhea-associated child mortality rates, implement rotavirus immunization programs [5]. Kenya adopted this recommendation in July 2014 [6, 7]. Since then, 2 doses of live-attenuated rotavirus vaccine (Rotarix), in addition to oral polio, pneumococcal conjugate, and pentavalent vaccines, have been recommended to children in Kenya, targeted at 6 and 10 weeks of life, as part of the routine child immunization program [8, 9]. It was estimated that introduction of this vaccine in Kenya would avert over 60 000 deaths and over 200 000 hospitalizations among children aged younger than 5 years during the first 20 years of introduction [10]. Following vaccine introduction, we used demographic and hospital data from 2 population-based surveillance sites in Kenya to estimate the impact of the program on rotavirus hospitalizations in children younger than 5 years.

## METHODS

### Geographical Location

The study was conducted in 2 regions with established demographic surveillance systems (DSSs): the Kilifi Health and Demographic Surveillance System (Kilifi HDSS) [11], which is located on the Kenyan coast, and the Kenya Medical Research Institute (KEMRI) and the Centers for Disease Control and Prevention (CDC) Health and Demographic Surveillance System (Siaya HDSS) [12] in rural western Kenya near Lake Victoria. Hospitalization, diarrhea, and rotavirus counts for children under 5 years of age were obtained from 2 government hospitals: Kilifi County Hospital located in the Kilifi HDSS and Siaya County Referral Hospital located in the Siaya HDSS.

### Vaccine Introduction and Coverage

Rotavirus vaccine was introduced into the childhood vaccination program in both Kilifi and Siaya counties in July 2014. Children who were aged 6 weeks or younger or born after the start of the rotavirus immunization program were eligible to receive 1 dose at 6 weeks and 1 dose at 10 weeks, although vaccine stock-outs in November and December 2014 kept some eligible children from receiving vaccine. There was no catch-up campaign in either Kilifi or Siaya.

In the Kilifi HDSS, vaccinations have been recorded comprehensively since 2008 through a network of approximately 30 health facilities (both private and public) (Supplementary Figure 1), as part of the Kilifi Vaccine-Monitoring Study (KiVMS) [13]. In the Siaya HDSS, over 20 health facilities administer vaccines to children (Supplementary Figure 2); since 2007, immunization data have been collected for children aged less than 5 years during household data collection rounds, which occur 2–3 times per year [12]. In addition, the vaccination status of children enrolled in the rotavirus surveillance at Siaya County Referral Hospital is recorded at the time of hospital admission. In both sites, card-confirmed vaccine information was captured by the field staff.

### Rotavirus Surveillance

In 2009, to prepare for vaccine introduction [12, 14], rotavirus surveillance was intensified at Siaya County Referral Hospital among children aged less than 5 years who presented with 3 or more loose stools within a 24-hour period and 1 or more vomiting episodes. In Kilifi, rotavirus surveillance was initiated in late 2009 in inpatient children aged less than 13 years with 3 or more loose stools within a 24-hour period. Parents of eligible children were briefed on the potential risks and benefits of the study before being asked to give consent by the study team. Stool samples were collected from eligible children whose parents provided voluntary informed consent and tested for rotavirus antigen by enzyme-linked immunosorbent assay, as described previously [14, 15].

Surveillance was interrupted by 12 strikes, including 7 before and 5 after rotavirus vaccine introduction, by different cadres of healthcare workers as indicated in Supplementary Table 1.

### Statistical Analysis

We used a controlled interrupted time-series analysis in which rotavirus-negative diarrhea hospitalizations acted as the control series to compare monthly rotavirus-associated hospitalizations (RVHs) before and after rotavirus vaccine introduction in children younger than 5 years of age. Specifically, RVHs from the period 54 months prior to (January 2010 to June 2014) were compared with hospitalizations from the period 36 months after (July 2014 to June 2017) vaccine introduction at each facility. For a month in which less than 100% of diarrhea cases were tested for rotavirus, the number of rotavirus-positive cases was scaled by multiplying the total number of diarrhea cases by the proportion who were rotavirus positive among those tested in that month.

A negative binomial regression was used to estimate the change in rotavirus hospitalizations in the first year (July 2014–June 2015), second year (July 2015–June 2016), and third year (July 2016–June 2017) post–vaccine introduction. The model included rotavirus-negative diarrhea (the control series) as an offset and calendar month and health worker industrial actions as covariates. Lag-1 Newey-West standard errors were used to adjust for autocorrelation [16]. A similar analysis was conducted for all-cause diarrhea using non–diarrhea hospitalizations as the control series.

For the analysis of rotavirus-positive diarrhea, a sensitivity analysis was conducted using a synthetic control [17–19] in the regression model instead of rotavirus-negative cases. The synthetic control was generated by weighting 3 conditions (rotavirus-negative diarrhea, pneumonia, malaria)—using the SYNTH [20] command in Stata—so that their sum closely resembled the prevaccine rotavirus-positive time series.

All statistical analyses were conducted using Stata13.1 software (Stata Corporation). All datasets, Stata files, and related documentation are available on an online data repository (https://doi.org/10.7910/DVN/PH4COG) [21].

### Ethics Review

Ethical approval to conduct this study was granted by the KEMRI Scientific and Ethics Review Unit (SERU; SSC 3049) and the CDC's institutional review board (protocol 6968).

## RESULTS

### Incidence and Seasonality

During the study period, there were 1513 and 1652 diarrhea hospitalizations in Kilifi County Hospital and Siaya County Referral Hospital, respectively. In Kilifi, 1142 (75.5%) of the diarrhea cases were tested, and among those tested, 315 (27.6%) were positive for rotavirus. In Siaya, 877 (53.1%) were tested and 197 (22.5%) were positive for rotavirus.

**Figure 1.**
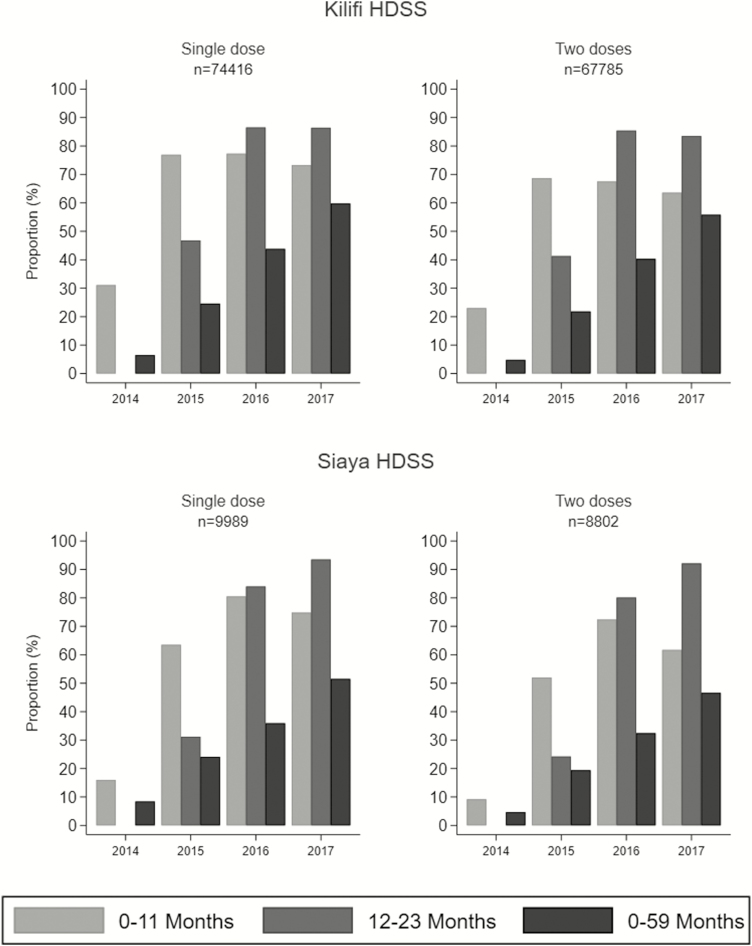
Proportion of children aged less than 5 years who had received 1 or 2 doses of rotavirus vaccine between 2014 and 2017 in Kilifi and Siaya demographic surveillance sites. Abbreviation: HDSS, Health and Demographic Surveillance System; n, number of children vaccinated.

The proportion of children living in the study areas who received rotavirus vaccine increased with time. Between 2014 (year of introduction) and 2017, the proportion of those aged less than 1 year with at least 1 dose increased from 31% to 73% in Kilifi and from 16% to 75% in Siaya; the proportion who were fully vaccinated (2 doses) in 2017 was 65% in Kilifi and 62% in Siaya. The highest coverage among infants was observed in 2016. Among children aged 12–23 months, the proportion with at least 1 dose increased from 0% to 86% in Kilifi and from 0% to 94% in Siaya; coverage with 2 doses in 2017 was 84% and 92%, respectively (Figure 1). The lowest proportions of vaccinated children were observed in 2014 across both sites due to vaccine roll-out in midyear and the unexpected stock-out. Among vaccinated children, the median age at receipt of the first dose was 6 weeks (interquartile range [IQR], 6–7 weeks) in Kilifi and 6 weeks (IQR, 6–7 weeks) in Siaya; median age at receipt of the second dose was 10 weeks (IQR, 10–13 weeks) in Kilifi and 10 weeks (IQR, 10–12 weeks) in Siaya.

**Figure 2.**
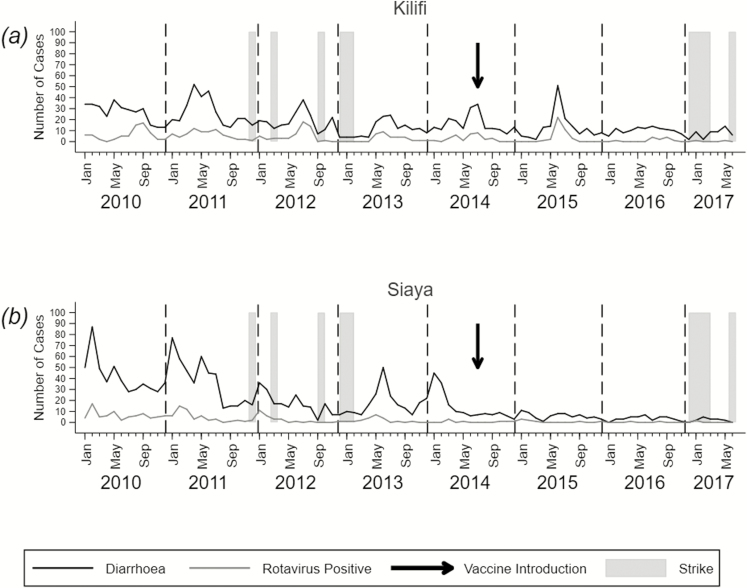
Monthly counts of diarrhea and rotavirus-positive cases in Kilifi (*A*) and Siaya (*B*) from January 2010 to June 2017. Strikes by health workers are indicated by gray shading, and the start of rotavirus vaccination in Kenya (July 2014) is indicated by an arrow.

Across the 2 DSSs, overall diarrheal illness and rotavirus-specific illness displayed seasonal patterns, which were dampened after the introduction of the vaccine (Figure 2). Siaya generally recorded higher diarrhea and rotavirus-associated diarrhea incidences compared with Kilifi in the prevaccination era (Tables 1 and 2). In Kilifi, the highest numbers of rotavirus cases were recorded from April/May to September/November of the same year (Figure 2A). In Siaya, the RVH season began earlier in the year, usually in January /February (Figure 2B). At both sites, there was a declining trend in annual incidence of diarrhea and RVHs that began prior to the rotavirus immunization program and continued following the introduction of vaccine (2015–2017). During the years affected by industrial actions of health workers (Supplementary Table 1) lower rotavirus incidence rates were recorded at both sites (Table 2).

**Table 1.** Monthly Diarrhea and Rotavirus Counts and Incidence Rates per 100 000 Children per Year by Site Prior to Rotavirus Vaccine Introduction (January 2010–June 2014)

	Kilifi						Siaya					
	Person-years	Diarrhea	Rotavirus (Observed)	Rotavirus (Scaled)	Diarrhea Incidence	Rotavirus Incidence	Person-years	Diarrhea	Rotavirus (Observed)	Rotavirus (Scaled)	Diarrhea Incidence	Rotavirus Incidence
January	20 041	90	19	22	449	110	8169	218	22	49	2669	600
February	20 041	86	12	16	429	80	8169	220	39	70	2693	857
March	20 041	103	15	19	514	95	8169	136	25	51	1665	624
April	20 041	113	21	29	564	145	8169	116	16	30	1420	367
May	20 041	125	22	29	624	145	8169	160	24	37	1959	453
June	20 041	158	37	47	788	235	8169	164	9	18	2008	220
July	16 055	118	38	52	735	324	6769	111	8	13	1640	192
August	16 055	78	39	46	486	287	6769	73	8	13	1078	192
September	16 055	65	25	33	405	206	6769	65	9	12	960	177
October	16 055	58	12	14	361	87	6769	70	7	11	1034	163
November	16 055	68	5	6	424	37	6769	73	6	12	1078	177
December	16 055	40	4	6	249	37	6769	81	9	19	1197	281

Rotavirus incidence rates are scaled as described in the Methods section.

**Table 2.** Annual Diarrhea and Rotavirus Incidence Rates per 100 000 Children Under 5 Years in Kilifi and Siaya, Kenya

Kilifi							Siaya					
Year	Population Estimate	Diarrhea	Rotavirus (Observed)	Rotavirus (Scaled)^a^	Diarrhea Incidence	Rotavirus Incidence	Population Estimate	Diarrhea	Rotavirus (Observed)	Rotavirus (Scaled)^a^	Diarrhea Incidence	Rotavirus Incidence
2010	47 746	319	70	93	668	195	21 885	500	78	124	2285	567
2011	48 083	323	74	90	672	187	20 953	446	53	107	2129	511
2012	48 412	213	56	70	440	145	19 947	201	26	50	1008	251
2013	48 414	140	31	42	289	87	18 439	218	21	45	1182	244
2014	47 835	196	31	40	410	84	16 803	162	7	13	964	77
2015	47 943	157	39	51	327	106	14 613	71	8	8	486	55
2016	46 109	116	12	13	252	28	12 806	39	2	2	305	16
2017	23 310	49	2	3	210	13	10 891	15	2	2	138	18

^a^Rotavirus incidence rates are scaled as described in the Methods section.

### Impact of Vaccine Using a Rotavirus-Negative Control Series

**Figure 3.**
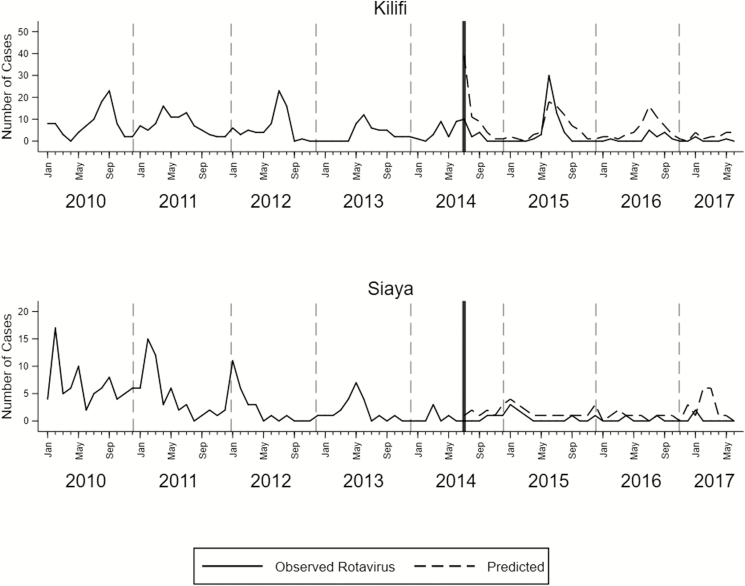
Monthly counts of rotavirus-positive cases (solid lines) and the number of cases (dashed lines) predicted by the model assuming no vaccine introduction. The vertical line indicates the time of vaccine introduction in Kenya (July 2014).

In Kilifi, we observed reduced rates of RVHs among Kilifi HDSS resident children younger than 5 years throughout the postvaccination period (Table 3 and Figure 3). When compared with rates from the prevaccine era, reductions were observed during the first year (57%; 95% confidence interval [CI], 8.0–80.0%), the second year (80%; 95% CI, 46–93%), and the third year (76%; 95% CI, 56–87%) (Supplementary Figure 3*a*). Similarly, in Siaya, there were reductions in the first year (59%; 95% CI, 20–79%), second year (82%; 95% CI, 61–92%), and the third year (81%; 95% CI, 7–96%) post–vaccine introduction (Supplementary Figure 3*b*).

**Table 3.** Incidence Rate Ratios Comparing Rotavirus and Diarrhea Incidence Pre– (January 2010–June 2014) and Post–Vaccine (July 2014–June 2017) Introduction in a Vaccine Impact Study in Kenya, 2010–2017

	Rotavirus			All-cause Diarrhea		
Post–Vaccine Period	IRR	95% CI	*P *value	IRR	95% CI	*P *value
Kilifi						
** **July 2014–June 2015	0.43	.20–.92	.031	0.59	.41–.84	.003
** **July 2015–June 2016	0.20	.07–.54	.002	0.52	.45–.60	<.001
** **July 2016–June 2017	0.24	.13–.44	<.001	0.54	.45–.66	<.001
Siaya						
** **July 2014–June 2015	0.41	.21–.80	.009	0.59	.48–.73	<.001
** **July 2015–June 2016	0.18	0.08–0.39	<.001	0.40	.28–.57	<.001
** **July 2016–June 2017	0.19	.04–.93	.040	0.38	.23–.62	<.001

See the Methods section for statistical details.Abbreviations: CI, confidence interval; IRR, incidence rate ratio.

### Impact of Vaccine on All-Cause Diarrhea

When examining trends in all-cause diarrhea using non–diarrhea hospitalizations as the control series, significant reductions were observed at both sites in post–vaccine introduction years (Table 3), although by a smaller magnitude compared with reductions in RVHs. In Kilifi, the impact of the vaccine on all-cause diarrhea increased from 41% (95% CI, 16–59%) in the first year to 48% (95% CI, 40–55%) and 46% (95% CI, 34–55%) in the second and third year, respectively (Supplementary Figure 3*e*). In Siaya, there was a 41% (95% CI, 27–52%) reduction in all-cause diarrhea in the first year followed by 60% (95% CI, 43–72%) in the second year and 62% (95% CI, 38–77%) in the third year (Supplementary Figure 3*f*).

### Impact of the Vaccine on Rotavirus-Associated Hospitalizations Using Synthetic Controls

The associated weights for each component of the synthetic control are shown in Supplementary Table 2 and the results of vaccine impact analysis in Supplementary Table 3 and Supplementary Figure 3. Vaccine impact estimates from this analysis are similar to those presented in Table 3, except that the estimate for the last year in Kilifi was slightly lower than the corresponding estimate in Table 3. In Kilifi, the impact of the vaccine was 67% (95% CI, 27–85%) in the first year, 86% (95% CI, 64–94%) in the second year, and 69% (95% CI, 45–83%) in the third year. Similarly, in Siaya, vaccine impact increased from 68% (95% CI, 33–84%) in the first year to 89% (95% CI, 68–96%) in the second year and 82% (95% CI, 8–96%) in the third year.

## DISCUSSION

We present results of an evaluation of the impact of rotavirus vaccine in Kenya using data from 2 population-based surveillance systems. Kenya introduced Rotarix vaccine into the national program as a 2-dose schedule (6 and 10 weeks of age). We observed that most vaccinated children at both sites received either 1 or both doses of the vaccine within 3 weeks of the recommended time of vaccination. We estimated the impact of the vaccine on RVHs using non–rotavirus hospitalizations and synthetic controls to control for any secular trends unrelated to the vaccine. Despite differences in the incidence of rotavirus disease in Kilifi and Siaya, as observed in this study and previous studies [15, 22], the impact of vaccination was similar at both sites. In the first year post–vaccine introduction, RVHs declined by close to 60% in both Kilifi and Siaya, and all-cause diarrhea declined by over 40% at both sites. In the second year of vaccination, RVHs further declined by over 80%, and this decline was sustained into the third post–vaccine introduction year with increasing coverage. Similarly, the positive impact of the vaccine on all-cause diarrhea increased in the second and third years to as high as 60% reduction, thus providing clear evidence of the substantial public health value of rotavirus immunization. These reductions suggest that vaccination of children aged less than 2 years, who are most likely to transmit rotavirus, optimizes direct and indirect protection against severe diarrheal infections.

Our estimates of rotavirus vaccine impact in the first year of vaccination are consistent with estimates from other African studies, which have ranged between 54% and 61% reduction for RVHs and between 43% and 48% for all-cause diarrhea hospitalizations [23, 24]. The same studies reported increasing impact in the second year of vaccination as was observed in our study.

Using the average of the 2013 Kilifi and Siaya rotavirus disease incidence estimates (166 per 100 000 per year) to represent disease incidence in Kenya in the absence of rotavirus vaccination, and assuming an under-5 population size of 6 million [25], an 80% vaccine impact equates to approximately 8000 rotavirus-related hospitalizations prevented per year in Kenyan children younger than 5 years. Over 20 years this corresponds to 160 000 hospitalizations prevented, which is similar to a previous estimate of 200 000 [10].

Our study was impacted by health worker industrial actions and a prevaccine secular trend in RVHs, possibly reflecting improved sanitation and hygiene [26]. To mitigate against these potential sources of bias we included the monthly rotavirus negative cases (or synthetic controls) as an offset term in our regression model. Additionally, we excluded the longest strike period (July–December 2017) from our analysis. An important assumption of this analysis is that the rotavirus-negative count reflects the counterfactual trend in rotavirus-positive cases that would have been observed in the absence of vaccination. Another limitation we considered was that only a fraction (75% in Kilifi and 53% in Siaya) of eligible diarrhea cases were tested for rotavirus, which represents another potential source of bias. To adjust for this, we imputed the number of rotavirus cases by multiplying the number of diarrhea cases by the fraction that tested positive. This imputation assumes that the fraction of rotavirus-positive cases is the same in those tested as it is in those who were not tested and may itself introduce bias, particularly if the fraction tested changes over time.

In conclusion, results from this study suggest that the burden of rotavirus and all-cause diarrhea declined substantially in Kenyan children in 2 regions of Kenya after rotavirus vaccine introduction. The estimates from this study represent total impact, indicating potential herd effects of the vaccine. Our estimates of vaccine impact on rotavirus and all-cause diarrhea were consistent across the 2 study sites and are consistent with findings from other African countries. These results contribute to the global estimates of rotavirus vaccine impact, especially in low- and middle-income countries, and will likely inform future decisions by policy makers on rotavirus vaccination.

## Supplementary Data

Supplementary materials are available at *Clinical Infectious Diseases* online. Consisting of data provided by the authors to benefit the reader, the posted materials are not copyedited and are the sole responsibility of the authors, so questions or comments should be addressed to the corresponding author.

ciz912_suppl_Supplementary_Figure_1

ciz912_suppl_Supplementary_Figure_2

ciz912_suppl_Supplementary_Figure3_revised

ciz912_suppl_Supplementary_Tables_and_Figures_Legends

## Notes

***Author contributions*.** G. P. O.: formal analysis, validation, writing—original draft and review and editing; data curation. C. B.: formal analysis, validation, writing—review and editing. I. A.: validation, investigation, methodology, writing—review and editing. S. K., M. N., R. O., B. O., B. E. O., G. B., J. B. O., C. O., J. J., J. M., C. T.: validation, investigation, writing—review and editing. J. E. T., Y. A., T. B.: investigation, methodology, writing—review and editing. U. D. P.: conceptualization, investigation, methodology, writing—review and editing. R. F. B.: conceptualization, funding acquisition, investigation, methodology, writing—review and editing. J. R. V.: conceptualization, funding acquisition, investigation, methodology, writing—review and editing. D. J. N.: funding acquisition, methodology, supervision (mentorship), writing—review and editing.

***Acknowledgments.*** The authors thank the study participants and clinical and research team members from both Kilifi and Siaya Health and Demographic Surveillance Systems who were involved at various stages of this study including sample collection and processing. This paper has been approved for publication by the director of the Kenya Medical Research Institute.

***Financial support.*** This study was funded by the GAVI (Global Alliance for Vaccines and Immunization) alliance under the Rotavirus Immunization Programme Evaluation in Kenya (RIPEK) Study, a collaboration between Emory University (USA); the Centers for Disease Control and Prevention in Atlanta, Georgia, USA; the Kenya Medical Research Institute (KEMRI)–Wellcome Trust Research Programme, Centre for Geographic Medicine Research-Coast, Kilifi; and the KEMRI Centre for Global Health Research, Kisumu, Kenya. The study was also supported by the Wellcome Trust (grant numbers 203077 and 102975).

***Potential conflicts of interest.*** The authors report no potential conflicts of interest. All authors have submitted the ICMJE Form for Disclosure of Potential Conflicts of Interest. Conflicts that the editors consider relevant to the content of the manuscript have been disclosed.
