# Supplementary figures and images for: Impact of the Introduction of Rotavirus Vaccine on Hospital Admissions for Diarrhea Among Children in Kenya: A Controlled Interrupted Time-Series Analysis
Source: Clin Infect Dis. 2019 Sep 23;70(11):2306–13. doi: 10.1093/cid/ciz912 (PMC7245159; doi:10.1093/cid/ciz912)

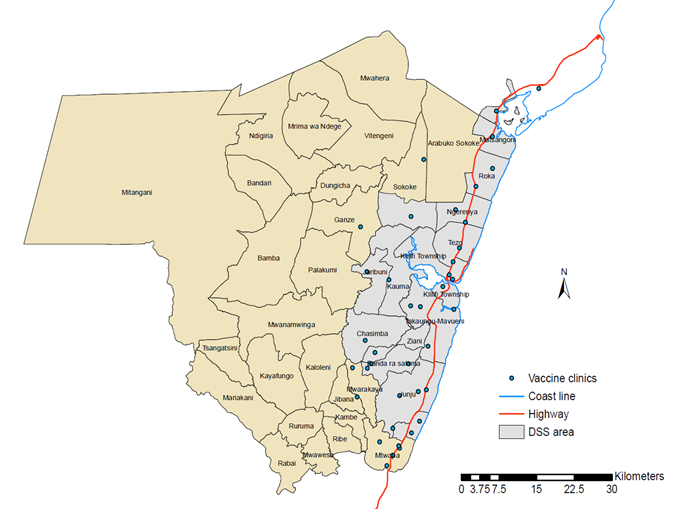

Supplement: ciz912_suppl_Supplementary_Figure_1 [file ciz912_suppl_supplementary_figure_1.png]

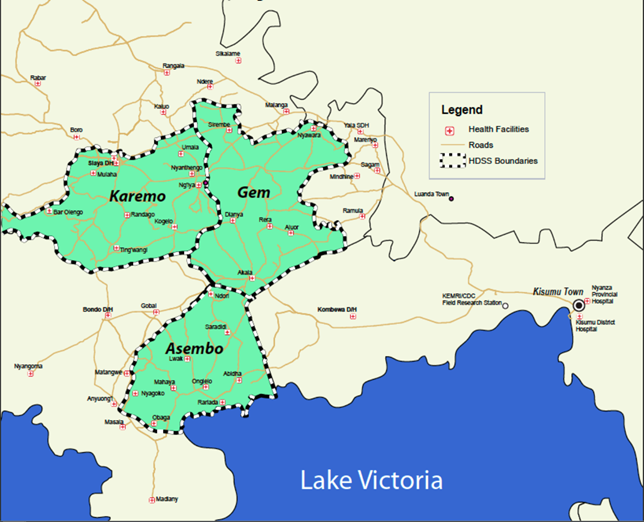

Supplement: ciz912_suppl_Supplementary_Figure_2 [file ciz912_suppl_supplementary_figure_2.png]

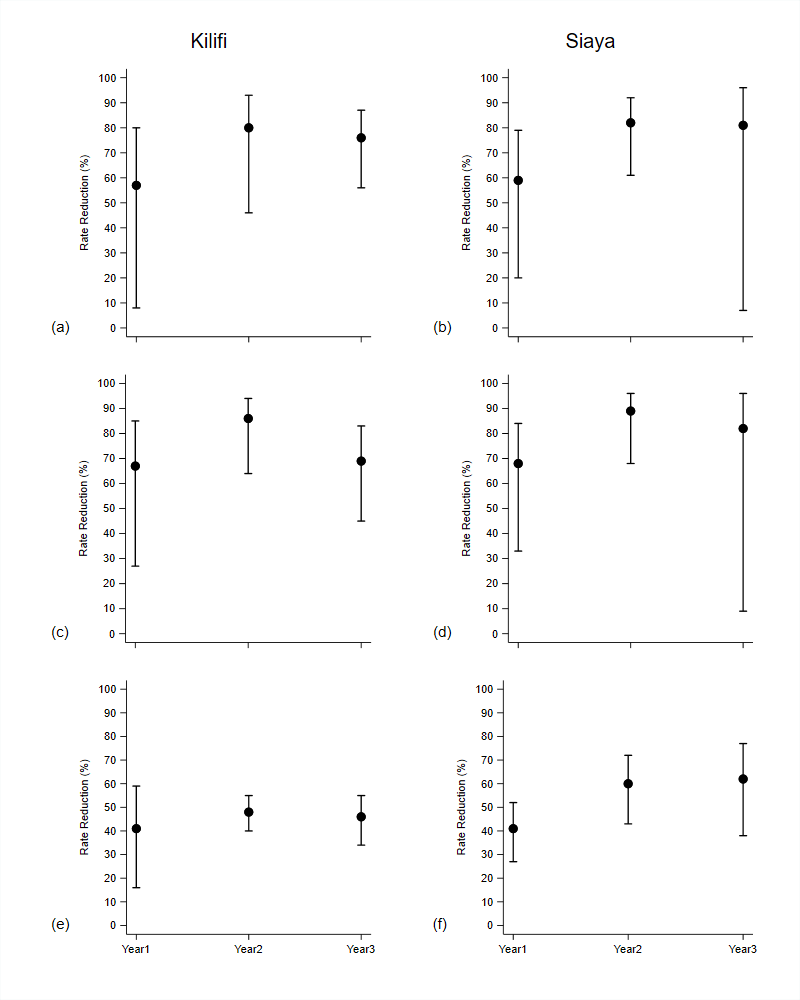

Supplement: ciz912_suppl_Supplementary_Figure3_revised [file ciz912_suppl_supplementary_figure3_revised.png]
