# Supplementary material for: Impact of the Introduction of Rotavirus Vaccine on Hospital Admissions for Diarrhea Among Children in Kenya: A Controlled Interrupted Time-Series Analysis
Source: Clin Infect Dis. 2019 Sep 23;70(11):2306–13. doi: 10.1093/cid/ciz912 (PMC7245159; doi:10.1093/cid/ciz912)
Supplement: ciz912_suppl_Supplementary_Tables_and_Figures_Legends [file ciz912_suppl_supplementary_tables_and_figures_legends.docx]

**APPENDIX 1: SUPPLEMENTARY TABLES**

**Supplementary** T**able 1: Nurses and doctors strike dates that affected ward operations at the paediatric wards of Kilifi county hospital and Siaya county referral hospital.**

| **Strike start date** | **Strike end date** | **Personnel** |
| --- | --- | --- |
|  |  |  |
| 5-Dec-2011 | 13-Dec-2011 | Doctors |
| 1-Mar-2012 | 16-Mar-2012 | Nurses |
| 13-Sep-2012 | 5-Oct-2012 | Doctors |
| 13-Dec-2012 | 13-Jan-2013 | Nurses |
| 16-Jan-2013 | 9-Feb-2013 | Nurses |
| 10-Dec-2013 | 23-Dec-2013 | Nurses |
| 10-Dec-2013 | 21-Dec-2013 | Doctors |
| 5-Dec-2016 | 23-Dec-2016 | Nurses |
| 5-Dec-2016 | 15-Dec-2016 | Doctors |
| 19-Dec-2016 | 14-Mar-2017 | Doctors |
| 2-Feb-2017 | 23-Feb-2017 | Nurses |
| 5-Jun-2017 | 3-Nov-2017 | Nurses |

**Supplementary Table 2: Weight assigned to each control condition/disease in synthetic RVA series by site**

|  | **Kilifi** | **Siaya** |
| --- | --- | --- |
|  |  |  |
| **Rotavirus Negative** | 0.470 | 0.224 |
| **Malaria** | 0.427 | 0.776 |
| **Pneumonia** | 0.103 | 0.000 |

**Supplementary Table 3: Incidence rate ratios (IRR) comparing RVA incidence pre and post vaccine introduction using synthetic controls.**

|  |  | **IRR** | **95% CI** | **p-value** |
| --- | --- | --- | --- | --- |
|  |  |  |  |  |
|  |  |  |  |  |
| **Kilifi** | **Jul14- Jun15** | 0.33 | 0.15-0.73 | 0.007 |
|  | **Jul15- Jun16** | 0.14 | 0.06-0.36 | <0.001 |
|  | **Jul16- Jun17** | 0.31 | 0.17-0.55 | <0.001 |
|  |  |  |  |  |
| **Siaya** | **Jul14- Jun15** | 0.32 | 0.16-0.67 | 0.002 |
|  | **Jul15- Jun16** | 0.11 | 0.04-0.32 | <0.001 |
|  | **Jul16- Jun17** | 0.18 | 0.04-0.91 | 0.038 |
|  |  |  |  |  |

**APPENDIX 2: SUPPLEMENTARY FIGURES**

**Supplementary Figure 1:** A map showing the geographical boundaries of the Kilifi Health and Demographic Surveillance System (KHDSS) and the health facilities participating in the vaccine monitoring system.

**Supplementary Figure 2:** A map showing the geographical boundaries of the Siaya health and demographic surveillance system (SHDSS) in western Kenya and health facilities that administer rotavirus vaccine.

**Supplementary Figure 3**: Relative reduction of rotavirus and all-cause diarrhoea hospitalisations. Graphs (a, c & e) are impact estimates from Kilifi and graphs (b, d & f) are estimates from Siaya. Graphs (a) and (b) show impact estimates of the vaccine on RVH using rotavirus negative control series while graphs (c) and (d) display impact estimates obtained using synthetic controls. Graphs (e & f) display impact impact on all-cause diarrhoea using all non-diarrhoea hospitalisations as the control series.

**APPENDIX 3: Model fitting**

The following model was fitted to data on the number of RVA cases per month:

$log(\lambda) =\log\left( C \right)+ \alpha+ \sum_{i=1}^{3} \beta_{i} X_{i}+ \sum_{i=2}^{12} \delta_{i} M_{i}+\sum_{i=1}^{7} \theta_{i} S_{i}$,

where

$$\lambda=expected number of RVA positive cases$$

$C=number of RVA negative cases$(the control)

$$X_{i}= indicator ith post vaccine period$$

$M_{i}=indicator ith calender month$

$$S_{i}=indicator ith industrial action$$

Note 1: The model was fitted using negative binomial regression, i.e., we assumed the number of RVA cases per month follows a negative binomial distribution.

Note 2: Newey-West standard errors were used to account for lag-1 autocorrelation.

Note 3: The estimates of vaccine impact, $\beta_{i}$, from this model are unbiased if, in the absence of an intervention, the RVA positive cases and RVA negative cases share a common trend.
